# Pre and postharvest characteristics of *Dahlia pinnata* var. pinnata, cav. As affected by SiO_2_ and CaCO_3_ nanoparticles under two different planting dates

**DOI:** 10.1016/j.heliyon.2023.e17292

**Published:** 2023-06-14

**Authors:** Mahmoud M. Kasem, Mohaned M. Abd El-Baset, Ahmed A. Helaly, El-Sayed A. EL-Boraie, Mashael Daghash Alqahtani, Abdulrahman Alhashimi, Abdelghafar M. Abu-Elsaoud, Amr Elkelish, Ahmed G. Mancy, Abdulrahman Alhumaid, Mostafa F. El-Banna

**Affiliations:** a Vegetable and Floriculture Department, Faculty of Agriculture, Mansoura University, Mansoura 35516, Egypt; b Vegetable and Floriculture Department, Faculty of Agriculture, Damietta University, New Damietta 34517, Egypt; c Department of Biology, College of Sciences, Princess Nourah Bint Abdulrahman University, P.O.BOX 84428, Riyadh 11671, Saudi Arabia; d Department of Botany and Microbiology, College of Science, King Saud University, P.O. Box 2455, Riyadh 11451, Saudi Arabia; e Department of Biology, College of Science, Imam Mohammad Ibn Saud Islamic University (IMSIU), Riyadh 11623, Saudi Arabia; f Botany and Microbiology Department, Faculty of Science, Suez Canal University, Ismailia 41522, Egypt; g Soils and Water Department, Faculty of Agriculture, Al-Azhar University, Nasr City 11884, Cairo, Egypt; h Department of Plant Production and Protection, College of Agriculture and Veterinary Medicine, Qassim University, Burydah 51452, Saudi Arabia; i Agricultural Botany Department, Faculty of Agriculture, Mansoura University, Mansoura 35516, Egypt

## Abstract

Agriculture faces many challenges because of climate changes. The nutrients present in nano-sized form improve plant productivity, especially when used at the appropriate planting time. Field experiments were conducted as a factorial experiment for evaluating two planting dates (20th September and 20th October)**,** foliar application with nanoparticles (NPs) including silica nanoparticles (SiO_2_-NPs) at 1.5 and 3 mM, calcium carbonate nanoparticles (CaCO_3_-NPs) at 5 and 10 mM and distilled water (control) on pre- and post-harvest characteristics of *Dahlia pinnata* var. pinnata Cav. The results indicate that the interactions during the late planting time (20th October) and exogenous applications of SiO_2_-NPs at 1.5 mM or CaCO_3_-NPs at 10 mM have improved plant growth including plant height, stem diameter, fresh and dry weights of plant, leaf area, inflorescence diameter, inflorescence stalk length, branches number, tuber numbers, inflorescences number on the plant, and the vase life. At the same time, insignificant differences appeared in the interaction during the planting dates and SiO_2_ or CaCO_3_ -NPs concentrations on inflorescence stalk diameter, total soluble solids, membrane stability index, maximum increase in fresh weight (FW), and Si and Ca contents. In addition, all exogenous applications of NPs at the late planting time promoted the plant growth characteristics like lignin %, cellulose %, inflorescence water content, change in FW, and total water uptake. Moreover, the controls through the two planting dates recorded the maximum change in water uptake and water loss values. In short, it can be recommended to use SiO_2_-NPs at 1.5 mM or CaCO_3_-NPs at 10 mM as a foliar application at the late planting time (20th October) for obtaining the optimum quantitative and qualitative parameters of *D. pinnata***.**

## 1 Introduction

Dahlia (*Dahlia pinnata* Cav.) is a magnificent tuberous root plant that widely utilized as a winter cut flower. It is a genus of a bushy perennial herbaceous plant native to Mexico and Central America comprising 42 species and more than 1,000 unique cultivars worldwide [1]. It has a lot of varieties with attractive colors, a fleshy brittle and liable-to-break stem, a wide range of plant heights (0.3–2.4 m), flower size (5.1–30 cm) and a long flowering period [2]. Dahlia tubers also have a low-calorie content, ranging from 180 to 193 kcal per 100 g of dry matter. The tubers' total dry matter has a rich fiber source (4.8%–11.1%), protein (∼16.39), lipids (∼0.15%) and carbohydrates (∼47.81%). Consequently, flowers are used as a fresh edible ingredient in salads, cream, cheese, and dips, and the tuberous roots are cooked in several traditional foods, especially in Mexico [3]. When compared to other countries, Egypt has a severe flower scarcity since dahlias have a short vase life (about 5–7 days), which may be a major factor in the decline of customer demand [4]. To meet the high demand for dahlia cut flowers, it is necessary to increase the qualitative and quantitative production aspects and the length of the flowering time.

The planting time plays a significant role in cut flowers' qualitative and quantitative characteristics through its impact on meteorological variables like temperature, rainfall, and humidity. According to Khan and coworkers, planting time intervals (between 15th October and 15th January) showed a significant effect on bulb production and flowering characteristics of tulips [5]. Additionally, Meena et al. demonstrated that most of the vegetative growth and flowering parameters of *Polyanthus tuberosa* plants were considerably affected by planting dates at intervals of 15 days from 15th April until 30th June [6]. In another investigation, *Freesia hybrida* planting on 1st of December resulted in greater inflorescence stem and floret head diameters, fresh inflorescence weight, and longer vase life compared with 15th of December [7].

Foliar application is a widely used technique for ornamentals nutrition. The thickness of cuticle layer and its composition, trichome type, and stomatal density affect how much absorption results from foliar spraying [8]. In this regard, NPs have been widely investigated in foliar nutrition of ornamentals and cut flowers. Nanoparticles (substances with particle sizes of less than 100 nm) exhibit unique physicochemical characteristics (surface area in particular), which enhances their availability to plants relative to their original form [9,10]. The plant cell wall surrounds the plasma membrane providing a tensile strength and a safeguard for the plant cell as well as allowing cells to develop a turgor pressure and improve plant tolerance and immunity [11,12].

SiO_2_-NPs have a very reactive surface-to-volume ratio to express greater efficiency than original Si form given their greater density of reactive sites [13,14]. SiO_2_-NPs have been documented in reducing the adverse effects of biotic and abiotic stress on several plants including marigolds [15], peppermints [16], tuberoses [17], and lilies [18]. Likewise, foliar application of SiO_2_-NPs recorded high efficacy relative to the soil application given its cost efficiency and environmental safety taking into consideration the potential translocation NPs to groundwater in case of soil application [19,20]. Several investigations illustrated that SiO_2_-NPs could enhance vegetative proliferation, improve physiological functionality and stimulate turgor pressure of plants [13,21,22]. Furthermore, applications of silica could extend the vase life of cut flowers through decreasing chlorophyll degradation, improving water balance, regulating CO_2_ transport inside cells and maximize nutrients uptake [[23], [24], [25]]. Furthermore, SiO_2_-NPs could maintain the peripheral link between lignin and carbohydrates, which aids the uprightness of the leaves and their durability against excessive nitrogen intake, lodging and low lighting conditions [26].

Calcium (Ca) plays certain roles in cell membranes, cell division and the sturdiness of cell walls through the formation of calcium pectate [27]. In addition, Ca combined with potassium (K) is important for cell function, permeability, dehydration, and the maintenance of turgor pressure, and significantly impacts the control of plant development and growth [28]. It integrates the structure of cell membranes by attaching to phospholipids, which stabilizes these layers [29]. The enhancement of photosynthetic pigment content, the management of antioxidant activity, water relations, accumulation of osmolytes, and nutritional balance have all been linked to Ca's beneficial effects on abiotic stress intolerance [30]. Additionally, spraying CaCO_3_-NPs showed pivotal roles in improving plant vigority, flowering, coloring, biotic and abiotic resistance and post-harvest quality characteristics [31]. On the other hand, Ca supplementations showed major roles in increasing the size and quantity of flower buds, reinforcing the flower’s stalk, and accelerating plant growth and development [32].

Nevertheless, there is a lack of research on the impact of SiO_2_-NPs or CaCO_3_-NPs on dahlia pre- and post-harvest characteristics, and this study was done to fill this gap of knowledge. Therefore, this research hypothesized that foliar application of SiO_2_-NPs and CaCO_3_-NPs could improve fleshy brittle, and liable-to-break stems and flowering growth characteristics of *Dahlia pinnata* var pinnata, Cav., as well as the post-harvest characteristics of the cut inflorescences if planted under the early and late planting dates of the Egyptian conditions.

## 2 Materials and methods

### 2.1 The experiment location and plant variety

**Fig. 1:**
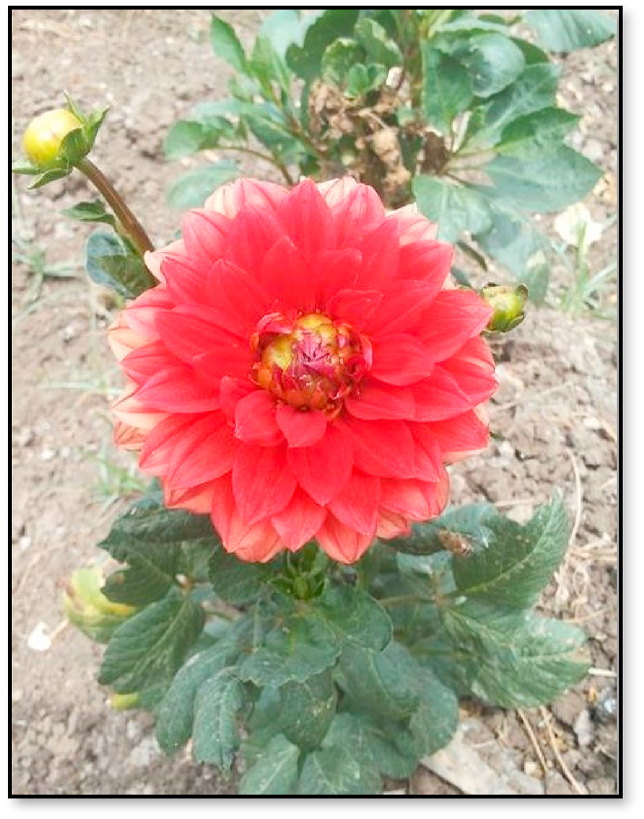
*Dahlia pinnata* var pinnata Cav., the examined variety.

A field experiment on Dahlia pinnata var pinnata Cav. was carried out at a private farm (31° 01' N latitude and 31° 25' longitude with an elevation of 6.95 m above sea level) at Gharbiya Governorate, Egypt, during two successive seasons 2019/2020 and 2020/2021 in a clay loamy soil (Fig. 1).

### 2.2 Plant source and planting procedure

**Fig. 2:**
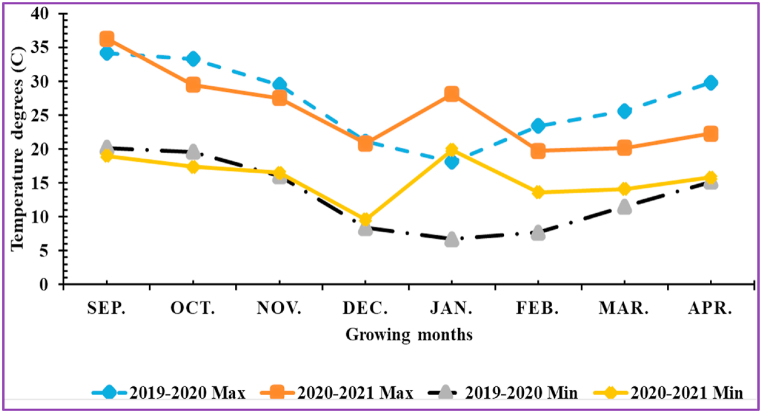
The experiment site's seasonal averages of maximum and minimum temperature during the growing seasons.

A uniform shape and size of *Dahlia pinnata*, var pinnata Cav. tubers were purchased from a commercial nursery in EL-Kanater El-Khaireia city, Qaluobiya Governorate, Egypt. Tubers were planted on two dates (20th September and 20th October) during both seasons in a plot size (2.4 × 4.6 m) consisting of three ridges at a length of 4 m for each with 65 cm between tubers and 80 cm between rows (18 plants) for each treatment. Tubers were planted taking into consideration the top position of buds in soil (5 cm from the surface layer). The experiment was preserved under natural conditions with ideal irrigation requirements. The following fertilization requirements were applied per feddan before the tubers were planted; 75 kg ammonium sulfate (NH_4_)_2_ SO_4_ + 75 kg calcium sulfate (CaSO_4_) + 250 kg superphosphate calcium [Ca(H_2_PO_4_)_2_ CaSO_4_] +100 kg sulfur (SO_4_) + 10 m^3^ farmyard manure. Representative soil samples were collected during both growing seasons from the rhizospheric layer (0–35 cm) before the experimental procedures. Samples were air-dried, crushed, and passed through a 2 mm sieve. A composite soil sample was prepared from these subsamples for analytical procedures. Soil physicochemical analyses were performed according to the standard methods [33]. Some physicochemical characteristics of the experimental soil are illustrated in Table 1. The monthly average values of the maximum and minimum temperature degrees during both seasons were recorded (Fig. 2). After tuber germination, all stems that appeared on the tuber were removed except for the strongest. To encourage these stems to branch, one pinching process was performed as soon as a pair or three leaves appeared (25–30 cm) by removing the growing terminal tip of the selected stems.

**Table 1:** Some physicochemical analyses of the experimental soil during the growing seasons.

Soil Characteristics			
Physical	Value	Soluble anions (meq/L)	Value
Fine sand	18.17	SO_4_^2-^	5.16
Coarse sand	7.68	HCO_3_^−^	3.15
Clay	39.48	CO_3_^2-^	0.00
Silt	34.67	CL^-^	3.51
Texture	Clay-loam	**Soluble cations (meq/L)**	
**Chemical**		Ca^2+^	4.04
Organic matter (%)	1.95	K^+^	5.31
CaCO_3_	4.52	Na^+^	1.13
EC (dS m^−1^ at 25°)	1.12	Mg^2+^	1.34
pH (1:2.5 w/v)	8.11	**Available micronutrients (ppm)**	
Total – N (%)	0.25	Cu	0.52
Available – P (ppm)	11.74	Zn	1.33
		Mn	1.48
		Fe	3.62
		Si (mg kg^−1^)	31.02

### 2.3 Experimental factors

An experiment was conducted to evaluate the effects of foliar application by two commercial nanoparticles: the first was SiO_2_-NPs (2 N 99% with a typical size of 10–80 nm, 60.09 molecular weight, spherical morphology, 1600 °C melting point, 2230 °C boiling point, 2533 kg/cm^3^ density, 59.9668 g/mol exact mass, and 59.967 Da monoisotopic mass), and the second was CaCO_3_-NPs (2 N 99% with a typical size of 15–40 nm, 100.09 molecular weight, and cubic or hexagonal morphology, 825 °C melting point, 2.93 kg/cm^3^ density, and specific surface area (SSA) of 30–60 m^2^/g range). The foliar concentrations of both NPs were selected based on those reported in literature [34,35]. SiO_2_ and CaCO_3_ -NPs were produced by the American Elements ® Company.

Three foliar applications of SiO_2_ and CaCO_3_ -NPs were applied in both seasons. The first foliar spraying was applied 25 days after the two planting dates and repeated twice at three weeks’ intervals (1.0 h before sunset). Foliar solutions were supplemented with 0.01% Tween-20 as a surfactant and sprayed until runoff (with approximately 50–100 ml for each plant). The pH value of foliar applications was adjusted up to 5.75 by adding KOH and HCl solutions (0.1 M, EL-Gomhoria Chemical Co., Egypt) since an acidic pH value is preferable for optimum foliar sprays of NPs.

### 2.4 Experimental design

A split-plot design with three replicates (the experimental unit was about 11 m^2^ comprising 18 plants) was laid out since the main plot contained the two planting dates (20th September and 20th October), and the subplot consisted of five foliar application treatments [T_1_, control (distilled water); T_2_, SiO_2_-NPs at 1.5 mM; T_3_, SiO_2_-NPs at 3 mM; T_4_, CaCO_3_- NPs at 5 mM; T_5_, CaCO_3_- NPs at 10 mM].

### 2.5 Data recorded

#### 2.5.1 Pre-harvest parameters

##### 2.5.1.1 Vegetative growth parameters

The vegetative growth parameters were recorded for plant height (cm), branches number plant^−1^, and stem diameter (mm) after 5 cm from the soil surface. Plant fresh weight (g), plant dry weight (g), leaf area (cm^2^), and tubers number plant^−1^, explaining that all the above attributes are calculated at the beginning of the flowering stage except for the tubers number plant^−1^ since it estimated at the end of the flowering stage and the experiment in both seasons.

##### 2.5.1.2 Flowering growth parameters

Flowering bud emergence [days after planting (DAP)] starting from the planting process until the emergence of the first floral bud on each treatment, inflorescence number/plant, inflorescence diameter (cm), inflorescence stalk length (cm), and inflorescence stalk diameter (mm) under the floral bud directly were estimated.

##### 2.5.1.3 Chemical parameters

Total chlorophyll was determined at the beginning of the flowering stage in the third leaves from the top of plants by using the spectrophotometric method in extracts prepared by submersing leaves tissue in dimethyl sulfoxide (DMSO) for 24 h. The total chlorophyll content was expressed in mg 100 g^−1^ FW according to Ref. [36], and the following equation was used for calculation: Total Chl = (20.2 OD_645_ + 8.02 OD_663_) V/1000 × W, where OD_645_ = absorbance at 645 nm; OD_663_ = absorbance at 663 nm; W = the sample mass (mg) and V = the solvent volume (ml). In addition, total anthocyanin content (mg 100 g^−1^ FW of petals) was calculated calorimetrically by the methodology of [37], since 0.5 g from fresh petals samples were mixed with 10 ml of ethanol-hydrochloric acid in a blender. The mixture was kept overnight in the refrigerator (4.0 °C.) and thereafter filtered over a Whatman filter paper (No. 1). The absorbance was measured using a spectrophotometer at a wavelength of 535 nm, and a blank solution of ethyl (95% v/v) acidic with hydrochloric acid (1%) was adopted. The total absorbance of the sample was calculated by the next equation.

$Total\;sample\;absorbance=\frac{Absorbance\;at\;535nm\times Volume\;prepared\;for\;calculation\times Total\;volume\times 100}{Volume\;of\;the\;extraction\;\left(ml\right)\times Weight\;of\;sample\;taken}$.

$$Total\;anthocyanin\;content\;in\;mg\;100\;g-1\;FW\;petals=\frac{Total\;sample\;absorbance}{98.2}$$

Total soluble solids percentage (TSS %) was calculated on the 2nd day from the beginning of inflorescence opening by using 0.25 cm^2^ from a piece of petals which was pressed subsequently in a drop of the gained extract which was placed into a hand refractometer according to Ref. [38]. Silicon (mg g^−1^ DW), calcium (mg g^−1^ DW), lignin (%), and cellulose (%) were determined under the inflorescence head by approximately 1.0 cm. Silicon content was determined by the methodology of Zhang and Dotson [39] and modified by Song et al. [40], as plant samples were washed before drying several times with tap water to clean dust and dirt, and then with distilled water three times to eliminate of surface impurities, then 0.5 g (DW) of samples were treated with acid mixtures of HF, HNO_3_ in a digestion tube. The digested samples were diluted using DI water for the colorimetric determination of Si. Acid-diluted samples were treated with ammonium molybdate, ascorbic acid and sodium nitrate before colorimetric determination at wavelength 660 nm by a T60 UV/VIS spectrometer. A standard curve of soluble silicon was prepared from a certified Sigma-Aldrich Si Standard Silicon Solution, TraceCERT, 1000 mg mL^−1^. The linearity was approved at R^2^ values ≥ 0.99. Calcium content was estimated using flame atomic absorption spectroscopy according to O’leary et al. with a calcium standard solution [41]. The inflorescence stalk lignin percentage was calculated according to the certified method of AOAC [42]. Cellulose (%) in the inflorescence stalk was determined by the acid chlorite method according to the standard method [43]. Lignin and cellulose contents were calculated gravimetrically as percentages weight of oven-dried samples. Samples were carried out in triplicate considering reproducibility protocols.

#### 2.5.2 Postharvest parameters

Representative samples of all treatments from inflorescences are harvested when two outer rows of the ray florets have opened. The basal part of the inflorescence stem end was pulsed in ethyl alcohol (95%) for 5 min to reduce latex accumulation, which could block the xylem vessels [44]. Inflorescences were placed in a glass graduated cylinders containing 100 ml of distilled water +1.5% sucrose as a carbon source +0.2 g L^−1^ silver nitrate as antibacterial and fungal agent in the vase solution for all treatments. Nine randomly selected inflorescences from each treatment were utilized for the calculation of all postharvest parameters. The postharvest characteristics were evaluated under 1000 lux from white fluorescent lamps with 16 h of photoperiod at room temperature of 25 ± 2 °C and relative humidity of 62%.

##### 2.5.2.1 Membrane stability index (MSI %) on the fifth day of shelf life

The MSI was measured based on the electrolyte leakage of petals according to Sairam et al. (1997) using the following formula: MSI = [1- (Ec_1_/Ec_2_)] × 100, where Ec_1_ is the electric conductivity kept at 40 °C for 30 min and Ec_2_ is the electric conductivity kept at 100 °C in boiling water bath for 15 min. Plant samples were washed several times with tap water, and then with distilled water three times to eliminate of any surface impurities and ions from the spraying.

##### 2.5.2.2 Vase life

Inflorescences were considered dead when more than 50% of the radial flowers had wilted [4].

##### 2.5.2.3 Inflorescence FW parameters

###### 2.5.2.3.1 Maximum increase in FW

The maximum increase in FW (g) shelf life^−1^ was determined by deducting the essential FW (the first day of holding cut inflorescences in preservative solution) from the heaviest FW of cut inflorescences, subsequently dividing the obtained result by the essential FW of the cut inflorescence.

###### 2.5.2.3.2 Change in fresh weight

Change in FW (g) fifth day^−1^ was estimated as the difference between weights of cut inflorescence (g) on the 5th day, and weights of the same cut inflorescence (g) at the beginning (initial weight), and hence dividing the obtained result by the essential FW of cut inflorescence [45].

##### 2.5.2.4 Water relationships

###### 2.5.2.4.1 Inflorescence water content

Inflorescence water content (g) was measured by subtracting the dry weight (DW) of the whole inflorescence from their corresponding FW and dividing the result by the inflorescence DW.

###### 2.5.2.4.2 Total water uptake ml shelf life^−1^

Total water uptake ml shelf life^−1^ was calculated by the totality of all absorbed preservative water through the vase life interval of the cut inflorescence according to Ref. [46].

###### 2.5.2.4.3 Change in water uptake ml fifth day^−1^

Estimation was done on the 5th day from the beginning of holding the cut inflorescences (after correction by the mean evaporation value) following the methodology of Hatamzadeh et al. with minor adjustments [47] by the following equation:

Change in water uptake ml 5th day^−1^ = $\frac{solution\;uptake\;on\;fifth\;day\;of\;the\;cut\;inflorescence}{fresh\;weight\;\left(g\right)\;on\;fifth\;day\;of\;the\;same\;cut\;inflorescence}$ x 100

###### 2.5.2.4.4 Change in water loss ml fifth day^−1^

Change in water loss ml fifth day^−1^ was estimated by the following equation:

Water loss = (water uptake at 5th day – (± change in FW at 5th day).

###### 2.5.2.4.5 Change in water balance ml fifth day^−1^

Change in water balance ml fifth day^−1^ was calculated by the following equation:

Water balance = change in water uptake at 5th day - change in water loss at 5th day.

### 2.6 Statistical analysis

The gathered data of both examined seasons were arranged for analysis of variance (ANOVA) in a split-plot design using [48] statistical CoHort software (Berkeley, CA, USA), and comparing among means was achieved using Duncan’s multiple range test at *p* ≤ 0.05 according to Ref. [49] and data were presented as means ± SE.

## 3 Results

### 3.1 Pre-harvest parameters

#### 3.1.1 Vegetative growth

**Fig. 3:**
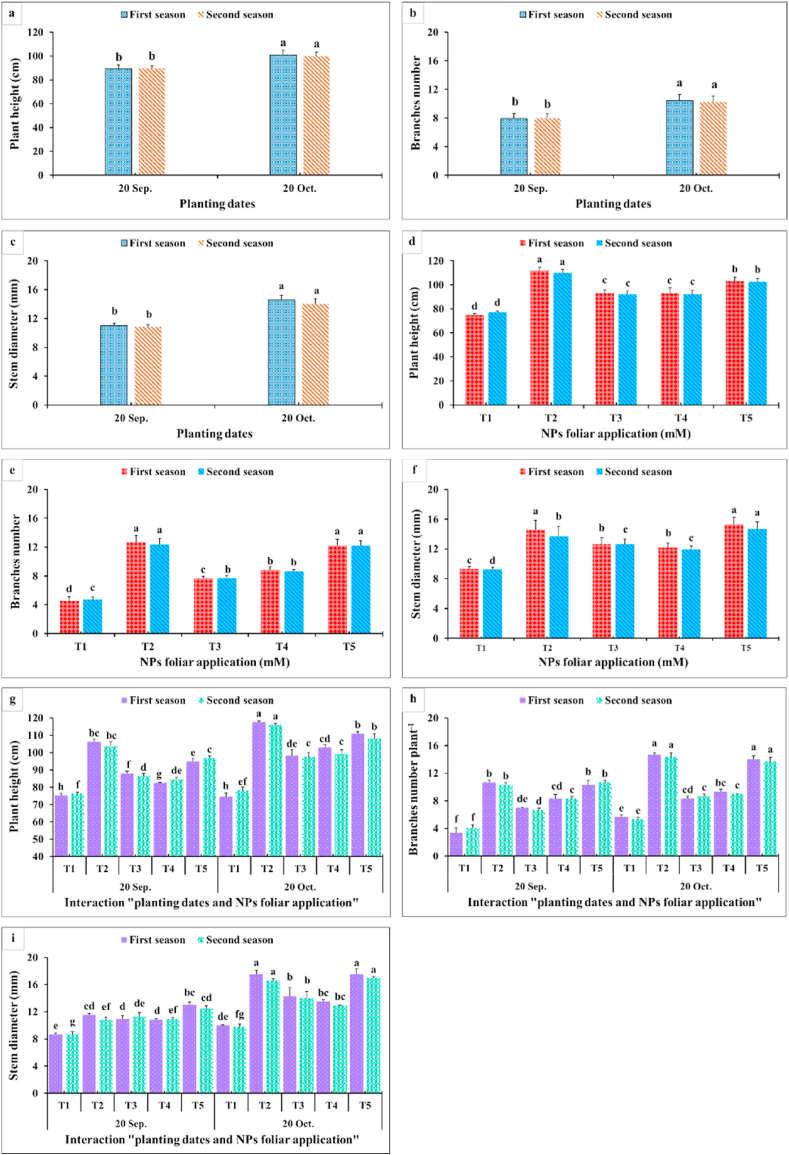
Impact of planting dates, NPs foliar application, and their interactions on plant height (a, d, g) (cm), branches number (b, e, h), and stem diameter (mm) (c, f, i) of *Dahlia pinnata* var pinnata Cav. during two seasons of 2019/2020 and 2020/2021. Different letters above columns at each season are significantly different (*p > 0.05*), (n = 3). T_1_, control (distilled water); T_2_, SiO_2_-NPs at 1.5 mM; T_3_, SiO_2_-NPs at 3 mM; T_4_, CaCO_3_- NPs at 5 mM; T_5_, CaCO_3_- NPs at 10 mM.

**Fig. 4:**
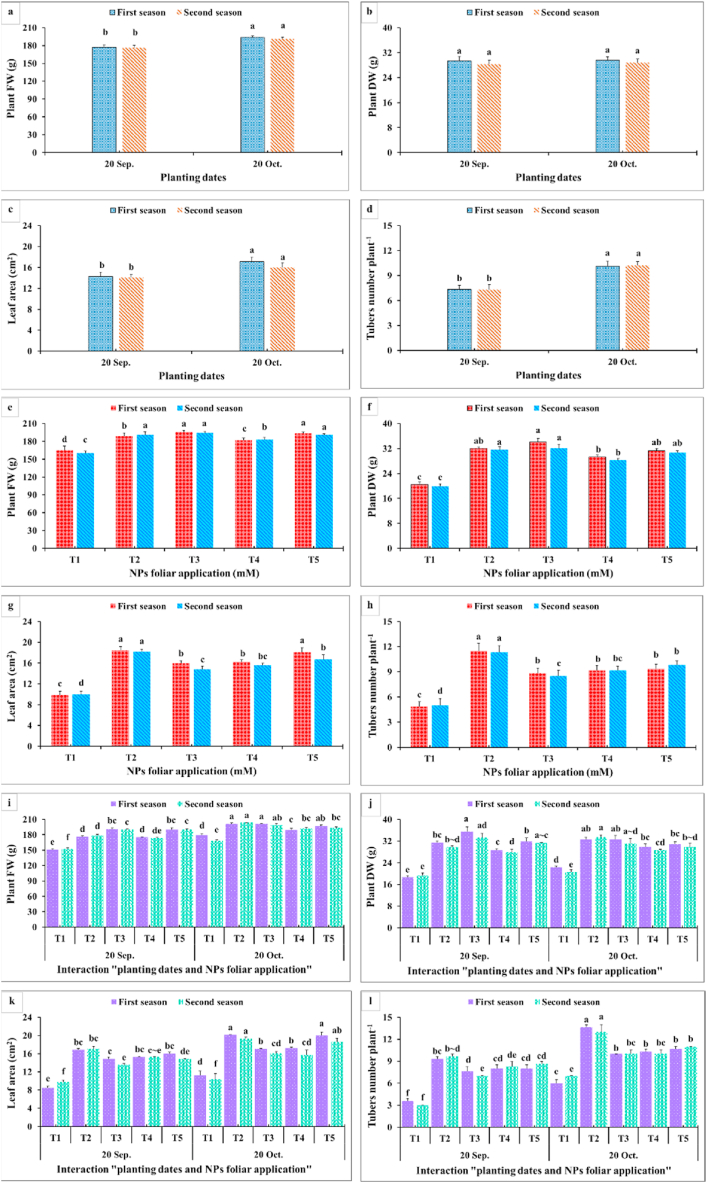
Impact of planting dates, NPs foliar application, and their interactions on plant FW (g) (a, e, i), plant DW (g) (b, f, j), leaf area (cm^2^) (c, g, k), and tuber numbers plant^−1^ (d, h, l) of *Dahlia pinnata* var pinnata Cav. during two seasons of 2019/2020 and 2020/2021. Different letters above columns at each season are significantly different (*p > 0.05*), (n = 3). T_1_, control (distilled water); T_2_, SiO_2_-NPs at 1.5 mM; T_3_, SiO_2_-NPs at 3 mM; T_4_, CaCO_3_- NPs at 5 mM; T_5_, CaCO_3_- NPs at 10 mM. FW (fresh weight); DW (dry weight).

Dahlia growth and vegetative characteristics parameters studied in the current research included plant height, branch number, stem diameter, FW, DW, leaf area, and tuber number per plant during two planting dates (Fig. 3, Fig. 4). In general, analysis of variance displayed significant effects of planting dates, NPs foliar application, and their interactions on these parameters. The late planting time (20th October) recorded the highest vegetative growth parameters. The early planting time (20th September) significantly reduced all the studied parameters except for the plant DW since an insignificant difference was found between the two planting dates. Among the tested NPs concentrations, dahlia plants sprayed with SiO_2_-NPs at 1.5 mM and CaCO_3_-NPs at 10 mM possessed the highest plant height (Fig. 3a,d), branches number (Fig. 3b,e), stem diameter (Fig. 3c,f), and leaf area (Fig. 4c,g), respectively, during both seasons. Moreover, the heaviest plant fresh (Fig. 4a,e) and dry weights (Fig. 4b,f) resulted from spraying SiO_2_-NPs at 1.5 or 3 mM and CaCO_3_-NPs at 10 mM during both seasons. In contrast, the control plants (plants sprayed with distilled water) recorded the lowest values for vegetative growth parameters. In addition, the late planting time (20th October) produced a higher tubers number plant^−1^ (Fig. 4 d, h) compared with the earlier planting time (20th September). Likewise, spraying SiO_2_-NPs at 1.5 mM and CaCO_3_-NPs at 10 mM produced the highest tubers number plant^−1^ during both seasons but applying SiO_2_-NPs at 1.5 mM was significantly higher than all NPs concentrations. As for the interaction, it was quite clear that the best results for plant height, branches number, and stem diameter of dahlia were associated with the combinations among 1.5 mM SiO_2_-NPs or 10 mM CaCO_3_-NPs and the late planting time (20th. October) (Fig. 4 g,h,i). Moreover, the plant’s fresh and dry weights were positively influenced by this interaction. In addition, spraying SiO_2_-NPs at 1.5 mM or CaCO_3_-NPs at 10 mM during the late planting time recorded the largest leaf area and the maximum tubers number plant^−1^ compared with all the other combinations. On the contrary, the interactions among all NPs foliar applications and the early planting time (20th September) presented lower values for all the vegetative parameters.

#### 3.1.2 Flowering parameters

**Fig. 5:**
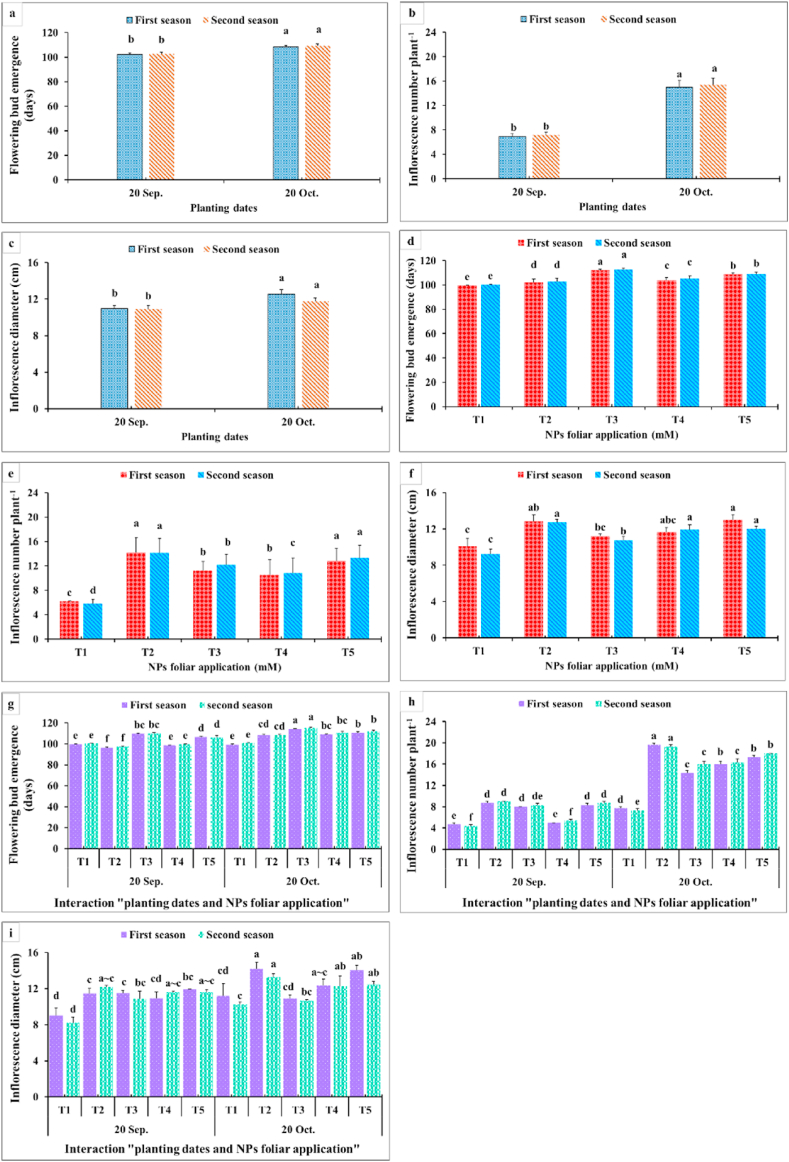
Impact of planting dates, NPs foliar application, and their interactions on flowering bud emergence (days) (a, d, g), inflorescence number plant^−1^ (b, e, h), and inflorescence diameter (cm) (c, f, i) of *Dahlia pinnata* var pinnata Cav. during two seasons of 2019/2020 and 2020/2021. Different letters above columns at each season are significantly different (*p > 0.05*), (n = 3). T_1_, control (distilled water); T_2_, SiO_2_-NPs at 1.5 mM; T_3_, SiO_2_-NPs at 3 mM; T_4_, CaCO_3_- NPs at 5 mM; T_5_, CaCO_3_- NPs at 10 mM.

**Fig. 6:**
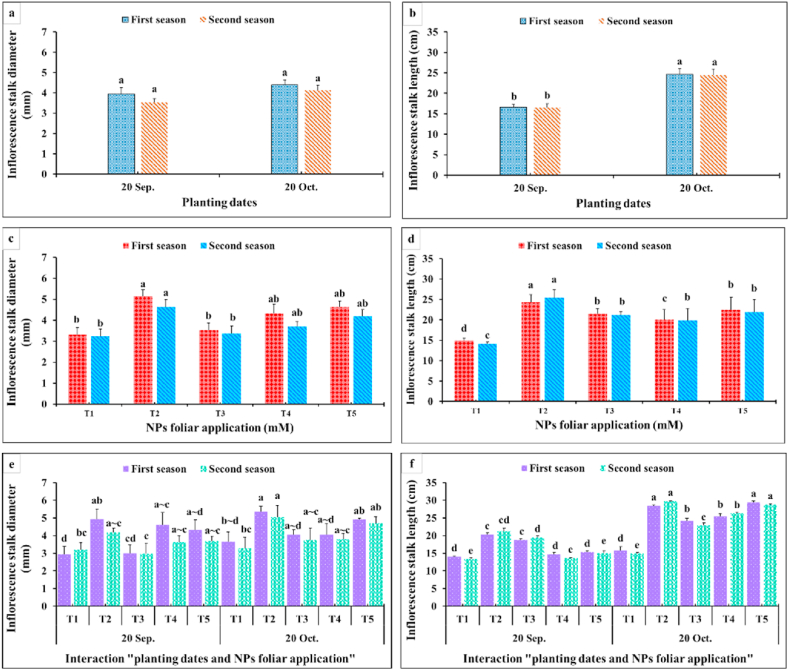
Impact of planting dates, NPs foliar application, and their interactions on inflorescence stalk length (cm) (a, c, e), and inflorescence stalk diameter (mm) (b, d, f) of *Dahlia pinnata* var pinnata Cav. during two seasons of 2019/2020 and 2020/2021. Different letters above columns at each season are significantly different (*p > 0.05*), (n = 3). T_1_, control (distilled water); T_2_, SiO_2_-NPs at 1.5 mM; T_3_, SiO_2_-NPs at 3 mM; T_4_, CaCO_3_- NPs at 5 mM; T_5_, CaCO_3_- NPs at 10 mM.

Flowering bud emergence, inflorescence number plant^−1^, inflorescence diameter, inflorescence stalk length (cm), and inflorescence stalk diameter (mm) (Fig. 5, Fig. 6) were significantly affected by the experimental factors. The lowest required days for flowering bud emergence were obtained from the earlier planting time of 20th September (Fig. 5). The evaluated NPs foliar application exhibited significant differences; since SiO_2_-NPs at 3 mM prolonged the required days for flowering bud emergence, while the control treatment (sprayed with distilled water) shortened this interval (Fig. 5a, d). The interaction between applying SiO_2_-NPs at 1.5 mM and the earlier planting time (20th September) decreased the required days for flowering bud emergence compared with all the other interactions except for the controls (Fig. 5g).

Noticeably, the planting time had a significant effect on the inflorescence number plant^−1^. The late planting time (20th October) produced the highest inflorescence number plant^−1^ compared with the earlier one (20th September), as it decreased the values of this parameter approximately to half (Fig. 5 b,e). Furthermore, spraying SiO_2_-NPs at 1.5 mM and CaCO_3_-NPs at 10 mM significantly gave the highest inflorescence number. Otherwise, the interaction between the late planting time (20th October) and spraying SiO_2_-NPs at 1.5 mM produced the highest values of inflorescence number plant^−1^ (Fig. 5h).

Similar trends for the inflorescence diameter were observed due to the main effects of planting time, NPs -foliar applications, and their interactions. The planting time had the upper hand in that respect since the late planting time (20th October) resulted in a higher inflorescence diameter than the earlier one (20th September) (Fig. 5c, f). In addition, CaCO_3_-NPs at 5 and 10 mM or SiO_2_-NPs at 1.5 mM improved the inflorescence diameter more than the control. Moreover, the highest significant values of inflorescence diameter were obtained as a result of interaction among SiO_2_-NPs at 1.5 mM or CaCO_3_-NPs at 5 and 10 mM under the late planting time (Fig. 5i). By looking at inflorescence stalk length as affected by the planting time, it was obvious from Fig. 6 that the late planting time significantly increased this parameter more than the earlier ones. Applying SiO_2_-NPs at 1.5 mM significantly produced the longest inflorescence stalk (Fig. 6a, c). Moreover, the interaction between the superior planting time (20th October) and foliar application of SiO_2_-NPs at 1.5 mM or CaCO_3_-NPs at 10 mM produced the tallest inflorescence stalk (Fig. 6e). On the other side, the planting time had an insignificant effect on inflorescence stalk diameter (Fig. 6b, d). In addition, foliar spraying of SiO_2_-NPs at 1.5 mM or CaCO_3_-NPs at 5 and 10 mM significantly produced superior inflorescence stalk diameter. In addition, it was obvious that the NPs foliar application had the upper hand in this interaction over the planting time (Fig. 6f).

#### 3.1.3 Chemical parameters

**Fig. 7:**
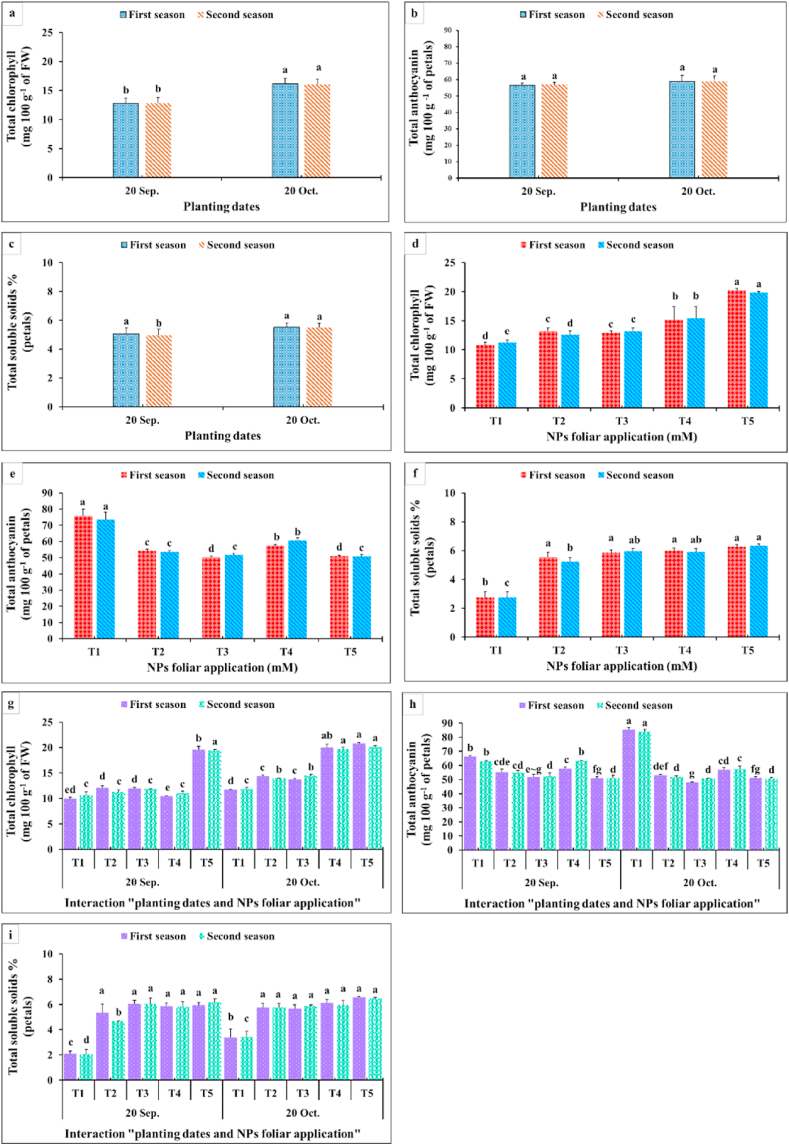
Impact of planting dates, NPs foliar application, and their interactions on total chlorophyll (mg 100 g^−1^ of FW) (a, d, g), total anthocyanin (mg 100 g^−1^ of petals) (b, e, h), and total soluble solids % (petals) (c, f, i) of *Dahlia pinnata* var pinnata Cav. during two seasons of 2019/2020 and 2020/2021. Different letters above columns at each season are significantly different (*p > 0.05*), (n = 3). T_1_, control (distilled water); T_2_, SiO_2_-NPs at 1.5 mM; T_3_, SiO_2_-NPs at 3 mM; T_4_, CaCO_3_- NPs at 5 mM; T_5_, CaCO_3_- NPs at 10 mM.

Concerning planting time, the dahlia tubers planted on 20th October recorded higher values of total chlorophylls in leaves, total soluble solids in petals and anthocyanin contents by about 25.27, 9.79 and 4.0%, respectively (Fig. 7 a,b,c,d,e,f). Consequently, the earlier planting time was not preferable as it might cause an adverse effect on the metabolic processes in plants. In addition, the maximum total chlorophyll contents were obtained when dahlia plants were sprayed with CaCO_3_-NPs at the maximum dose of 10 mM. Moreover, applying the SiO_2_-NPs or CaCO_3_-NPs, especially when their concentrations were increased, showed lower values for anthocyanin contents compared with the controls (Fig. 7 a,d).

**Fig. 8:**
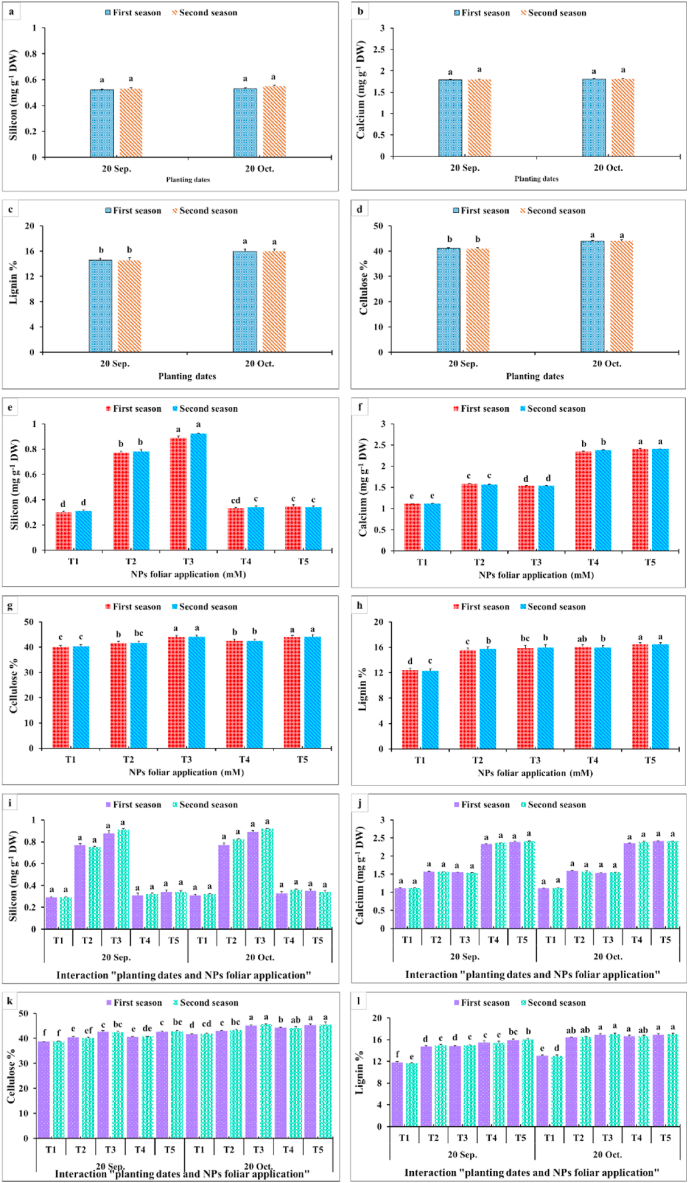
Impact of planting dates, NPs foliar application, and their interactions on silicon (mg g^−1^ DW) (a, e, i), calcium (mg g^−1^ DW) (b, f, i), lignin % (c, g, k), and cellulose % (d, h, i) of *Dahlia pinnata* var pinnata Cav. during two seasons of 2019/2020 and 2020/2021. Different letters above columns at each season are significantly different (*p > 0.05*), (n = 3). T_1_, control (distilled water); T_2_, SiO_2_-NPs at 1.5 mM; T_3_, SiO_2_-NPs at 3 mM; T_4_, CaCO_3_- NPs at 5 mM; T_5_, CaCO_3_- NPs at 10 mM. DW (dry weight).

As for the interaction between the planting time and the NPs foliar application on these parameters, it was clear that the maximum total chlorophyll contents were produced when dahlia was planted on 20th October and sprayed by CaCO_3_-NPs at 5 or 10 mM during both seasons (Fig. 7g). Differently, insignificant differences were obtained from the interaction among the planting time and the NPs foliar application on the total soluble solids% (Fig. 7i). However, all interactions were still significantly higher than the control ones. The superior total soluble solids percentages were produced from the interaction between the late planting time (20th October) and CaCO_3_-NPs at 10 mM (Fig. 7i). Data in Fig. 8a, b,e, f showed an insignificant difference between the two examined planting dates on the contents of silicon and calcium. In addition, the late planting time (20th October) produced higher significant values for lignin and cellulose percentages compared with the earlier one (Fig. 8 c,h,d,g). On the other hand, applying SiO_2_-NPs at 3 mM produced the highest silicon content (Fig. 8 a,e). The maximum calcium content was gained from spraying CaCO_3_-NPs at 10 mM. In addition, spraying 10 mM CaCO_3_-NPs recorded the highest lignin and cellulose percentages (Fig. 8 c,h,d,g). It was noticeable from Fig. 8 that an insignificant difference appeared among all interactions between planting time and NPs foliar applications on the silicon and calcium content. Likewise, spraying CaCO_3_-NPs at 5 and 10 mM or SiO_2_-NPs at 3 mM on dahlia which was planted at the late planting time produced superior lignin and cellulose percentages in the inflorescence stalks.

### 3.2 Post-harvest parameters

**Fig. 9:**
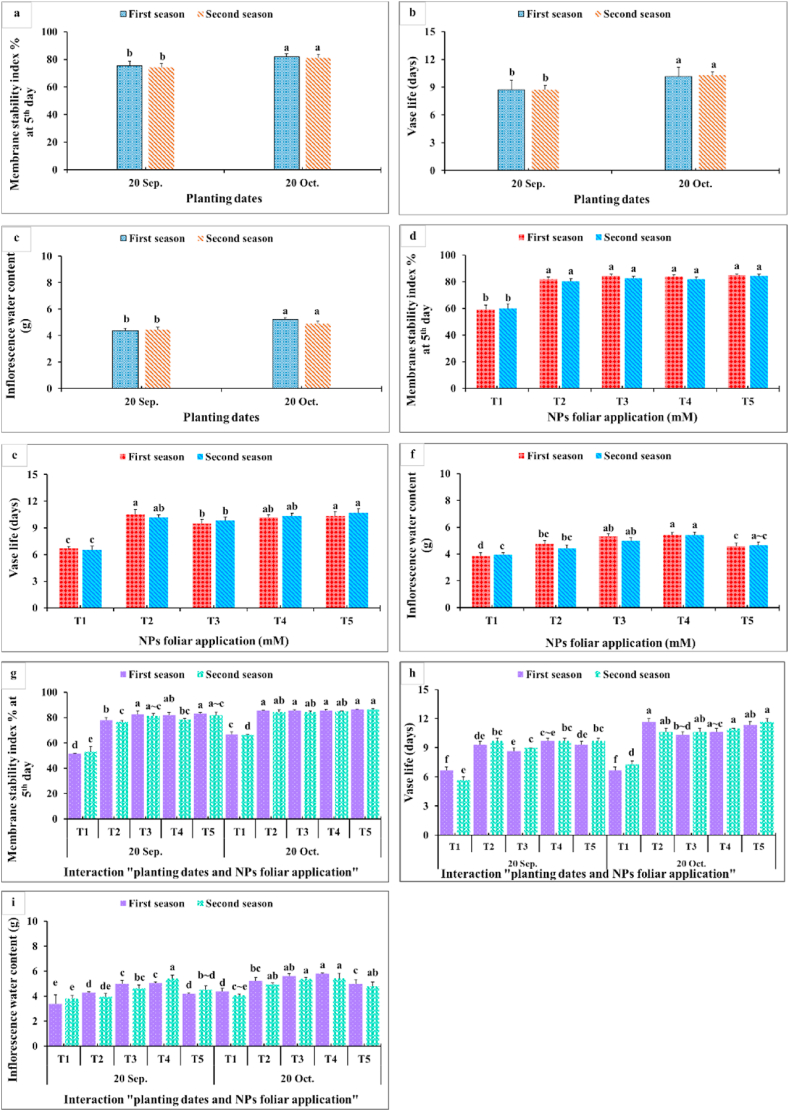
Impact of planting dates, NPs foliar application, and their interactions on membrane stability index % at 5th day (a, d, g), vase life (days) (b, e, h), and inflorescence water content (g) (c, f, i) of *Dahlia pinnata* var pinnata Cav. during two seasons of 2019/2020 and 2020/2021. Different letters above columns at each season are significantly different (*p > 0.05*), (n = 3). T_1_, control (distilled water); T_2_, SiO_2_-NPs at 1.5 mM; T_3_, SiO_2_-NPs at 3 mM; T_4_, CaCO_3_- NPs at 5 mM; T_5_, CaCO_3_- NPs at 10 mM.

**Fig. 10:**
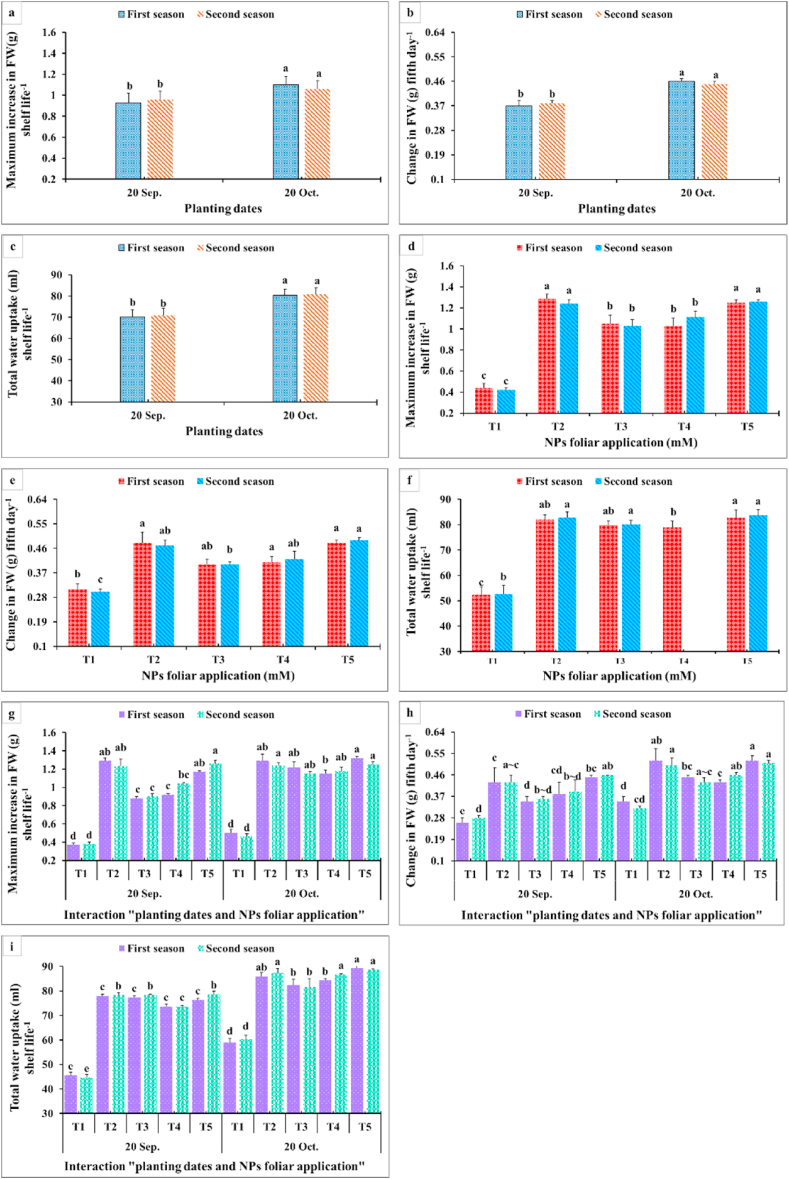
Impact of planting dates, NPs foliar application, and their interactions on the maximum increase in FW (g) shelf life^−1^ (a, d, g), change in FW (g) fifth day^−1^ (b, e, h), and total water uptake (ml) shelf life^−1^ (c, f, i) of *Dahlia pinnata* var pinnata Cav. during two seasons of 2019/2020 and 2020/2021. Different letters above columns at each season are significantly different (*p > 0.05*), (n = 3). T_1_, control (distilled water); T_2_, SiO_2_-NPs at 1.5 mM; T_3_, SiO_2_-NPs at 3 mM; T_4_, CaCO_3_- NPs at 5 mM; T_5_, CaCO_3_- NPs at 10 mM. FW (fresh weight).

**Fig. 11:**
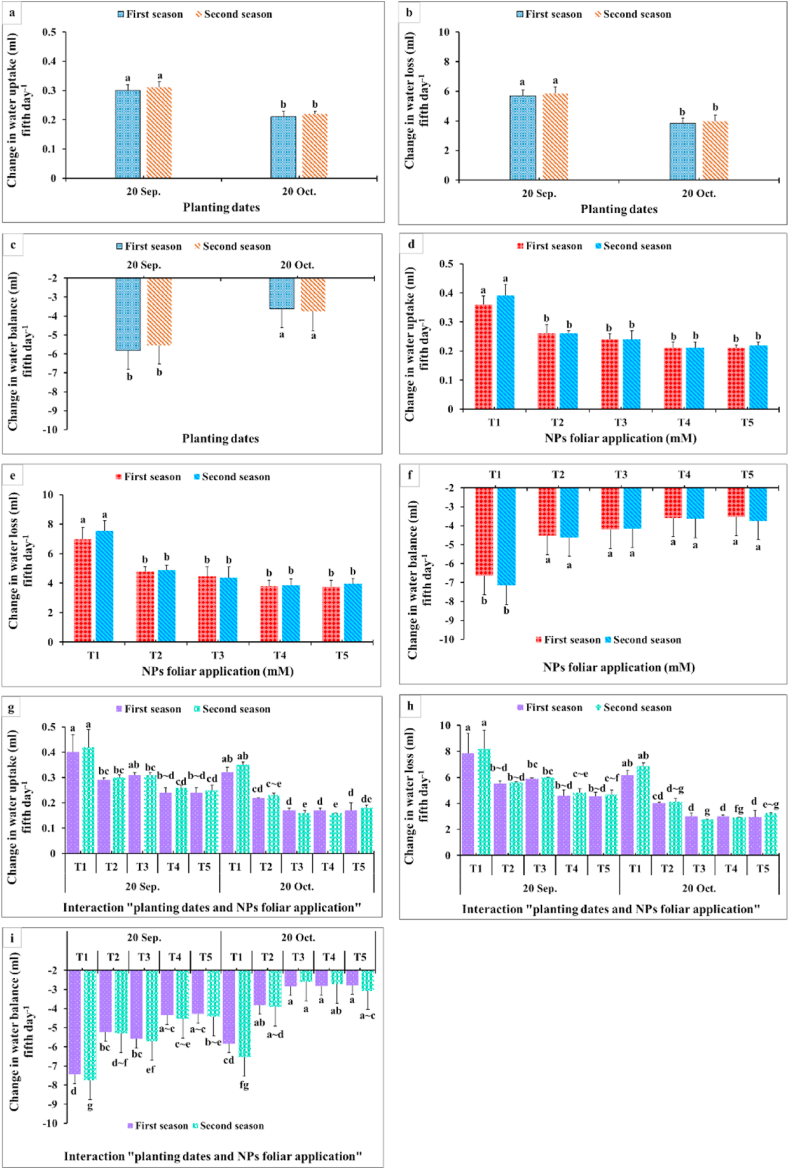
Impact of planting dates, NPs foliar application, and their interactions on change in water uptake (ml) fifth day^−1^ (a, d, g), change in water loss (ml) fifth day^−1^ (b, e, h), and change in water balance (ml) fifth day^−1^ (c, f, i) from shelf life of *Dahlia pinnata* var pinnata Cav. during two seasons of 2019/2020 and 2020/2021. Different letters above columns at each season are significantly different (*p > 0.05*), (n = 3). T_1_, control (distilled water); T_2_, SiO_2_-NPs at 1.5 mM; T_3_, SiO_2_-NPs at 3 mM; T_4_, CaCO_3_- NPs at 5 mM; T_5_, CaCO_3_- NPs at 10 mM.

Results in Fig. 9, Fig. 10, Fig. 11 showed that membrane stability index percentage, vase life (days), inflorescence water content, maximum increase in FW shelf life^−1^, change in FW (g) fifth day^−1^, total water uptake (ml) shelf life^−1^, change in water uptake (ml) fifth day^−1^, change in water loss (ml) fifth day^−1^, and change in water balance mm fifth day^−1^ significantly were affected by the planting time, NPs foliar applications, and their interactions. Since the maximum values of these parameters were obtained from the late planting time (20th October), except for the water relations (change in water uptake, water loss, and water balance). All NPs foliar treatments enhanced these parameters compared to the controls. All SiO_2_ and CaCO_3_ NPs foliar applications gave higher significant values for membrane stability index compared with the control, while the longest vase life was obtained from applying SiO_2_-NPs at 1.5 mM (Fig. 9 a,d). Moreover, spraying SiO_2_-NPs at 3 mM or CaCO_3_-NPs at 5 mM produced the maximum inflorescence water content (Fig. 9 c,f). The maximum increase in FW (g) shelf life^−1^ was obtained from dahlia plants sprayed with either 1.5 mM SiO_2_-NPs or 10 mM CaCO_3_-NPs (Fig. 9 b,c). In most of the cases, insignificant differences appeared between all SiO_2_-NPs and CaCO_3_-NPs concentrations on change in FW and the total water uptake, change in water uptake, water loss, and water balance (Fig. 10).

Data presented in Fig. 9 revealed that the late planting time (20th October) with applying all SiO_2_-NPs or CaCO_3_-NPs foliar applications produced the highest membrane stability index (Fig. 9 a,d). Moreover, the longest vase life (Fig. 9 b,c) and inflorescence water content values (Fig. 9 c,f) were obtained from dahlia plants sprayed with most of SiO_2_-NPs or CaCO_3_-NPs concentrations during the late planting time (20th October). This means that the planting time had the upper hand over the NPs foliar applications in that respect. Furthermore, the maximum increase in FW (g) shelf-life^−1^ values were gained from applying 1.5 mM SiO_2_-NPs or 10 mM CaCO_3_-NPs at the two examined planting dates (Fig. 10g). In addition, the highest values for change in FW fifth day^−1^ and the total water uptake shelf life^−1^ were obtained from the interaction between applying 1.5 mM SiO_2_-NPs or 5 and 10 mM CaCO_3_-NPs and the late planting time (20th October) (Fig. 10h and i). Additionally, changes in water uptake, water loss, and water balance on the fifth day significantly responded positively to the planting time and the NPs foliar application (Fig. 11). The late planting time (20th October) decreased the change in water uptake and water loss compared with the control and improved the water balance (Fig. 11 a,b). Moreover, all SiO_2_-NPs and CaCO_3_-NPs foliar applications decreased the change in water uptake and water loss more than the control and preserved higher water balance values (Fig. 11 d,e). It was quite clear that interaction between the planting time and SiO_2_-NPs or CaCO_3_-NPs foliar application was different from most of the other studied parameters since applying 1.5 mM SiO_2_-NPs and 5 or 10 mM CaCO_3_-NPs on dahlia plants which were planted at the earlier date (20th September) decreased the change in water loss (Fig. 11 h). Finally, the highest change in water balance values was observed with dahlia cut inflorescence which was planted at the late date (20th October) and sprayed with all SiO_2_-NPs or CaCO_3_-NPs concentrations (Fig. 11 c,f).

## 4 Discussion

### 4.1 Impact of planting date on pre- and post-harvest parameters

As observed in Fig. 3, Fig. 4, all growth and vegetative characteristics of *Dahlia pinnata* var pinnata Cav were seen to be highest at the late planting time (20th October) compared with the earlier one (20th September). These results may be due to the influence of environmental conditions, especially the temperature degrees between the two planting dates [50], since the mean temperature degree through the earlier planting time during both seasons was 34 and 36 °C, respectively, with approximately 5 higher temperature degrees than the late planting time (Fig. 2). This increase in temperature during the early planting time may increase the demolition processes that take place in the plant, especially at night, which results in a decrease in most of the vegetative, flowering, and chemical characteristics of the dahlia plant. According to similar tendencies [51], made it abundantly evident that planting dates had a substantial impact on the growth and development of *Tagetes minuta* as seen by the change in the growth phase and the partitioning of the aerial biomass. According to Ref. [52], the summer savory production was significantly impacted by the sowing dates (10th April, 25th April, and 10th May). Our findings are similar to different studies, since the growth and vegetative characteristics were influenced by the planting dates of *Mentha arvensis* [53], and *Marrubium vulgare* [54]. In addition, the current results revealed that the earlier planting time (20th September) reduced the required days for flowering bud emergence by 6.06 and 6.67 days during both seasons compared with the late planting time. On the contrary, the earlier planting date (20th September) reduced the inflorescences number plant^−1^ by 53.80 and 53.70%, inflorescence diameter by 12.52 and 7.55%, inflorescence stalk length by 32.68 and 32.54%, and inflorescence stalk diameter by 10.23 and 14.32% during both seasons compared with the late planting time. Similar trends were observed in another studies on tulips [5], *Polyanthus tuberosa* [6], and *Freesia hybrida* [7]. Moreover, the late planting time (20th October) increased the chemical constituents like total chlorophylls by 25.98 and 24.53%, total anthocyanin by 4.35 and 3.47%, total soluble solids by 8.69 and 10.89%, silicon by 1.92 and 3.77%, calcium by 1.12 and 0.56%, lignin by 9.90 and 9.88%, and cellulose by 6.95 and 7.51% than the earlier planting time (Fig. 7, Fig. 8). The previous findings of flowering and chemical parameters are linked with the environmental conditions as discussed previously, and what confirms this is the exposure of the plant to high temperatures on the earlier planting time, which accelerated the entry of the plants to the flowering stage and reduced all the flowering parameters compared to the late date. These findings were in line with [55] on China aster [56]. In addition, suitable planting dates could positively stimulate flowering characteristics by supplying enough food gained from the photosynthesis process [57].

Furthermore, the planting time influenced all the studied post-harvest parameters, as shown in Fig. 9, Fig. 10, Fig. 11. In particular, the late planting time (20th October) increased the membrane stability index % by 7.97 and 8.79%, vase life by 13.82 and 14.99%, inflorescence water content by 15.79 and 9.36%, maximum increase in FW by 15.45 and 9.43%, change in FW by 19.56 and 15.55%, total water uptake by 12.46 and 12.60%, and water balance by 61.04 and 47.21% compared with the earlier planting time (20th September) during both seasons, respectively. On the contrary, the earlier planting time (20th September) increased the change in water uptake by 30.00 and 29.03% and the change in water loss by 32.57 and 31.79% compared with the late date. In another study on *Freesia hybrida,* staggering the planting time from the 1st of December to the 15th of the same month significantly reduced the flowers’ vase life, and the suitable planting time (1st December increased the inflorescence stem and floret head diameters, and fresh inflorescence weight [7]. Furthermore, the fact that the late planting time was during a time when climatic conditions, especially the temperature, were not as scorching resulting in less utilization of synthesized photosynthates carbohydrates, might be an explanation for the positive impact on most of the post-harvest characteristics. This finding was consistent with that of [58] on chrysanthemums.

### 4.2 Impact of SiO_2_-NPs and CaCO_3_-NPs on pre- and post-harvest parameters

The current research indicated that all foliar applications of SiO_2_-NPs and CaCO_3_-NPs had positive effects in improving pre- and post-harvest parameters compared with the control (distilled water) since spraying SiO_2_-NPs at 1.5 mM or CaCO_3_-NPs at 10 mM gained the highest vegetative and flowering characteristics. These findings agreed with those of [59], who found that treatment with 500 g L^−1^ SiO_2_-NPs increased the plant height, root diameter, main root length, and the number of lateral roots of Changbai larch seedlings by 5.42%, 30.7%, 14%, and 31.6%, respectively, compared to control. These results were supported by the findings of [60], which showed that SiO_2_-NPs might interact directly with plants and influence their physiology and morphology in a diverse range of ways, including providing structural color to the plants and enhancing crop productivity. In addition, Si-NPs were reported to marginally improve flowering in comparison to bulk Si or the control [61], making photosynthetic activity conceivable [62], affecting Si absorption, transport, and accumulation, and promoting cell wall lignification [63]. On banana plants grown *in vitro* with SiO_2_-NPs [64], there was noted a rise in growth parameters such as plant height, leaf number, FW, and DW because of enlarging the leaf area that promotes photosynthetic activity. The current results were in similar trends with the findings of [15] on marigolds [16], on peppermints [17], on tuberoses, and [18] on liliums. The positive effects of SiO_2_-NPs had been discussed regarding its ability to improve vegetative growth through increasing the total soluble protein content, and accelerating the photosynthesis process by accumulating amino acids and nutrients [21]. In addition, SiO_2_-NPs enhance water usage efficacy and leaf relative water content [13], and decrease transpiration rates [22].

Furthermore, calcium also appears to be crucial in enhancing physiological mechanisms by strengthening the cell wall and lowering the synthesis of ethylene in addition to being a component of the cell wall [65]. The current study shows that the usage of SiO_2_-NPs and CaCO_3_-NPs caused a drop in anthocyanin levels because, under adverse conditions, plants produce more anthocyanin to protect their photosynthetic system. Therefore, it appears that after applying CaCO_3_-NPs and SiO_2_-NPs, the plants' condition improved, lowering their anthocyanin concentration [66]. Additionally, calcium is crucial for a wide range of physiological and biochemical aspects of plant development, including the action of the rubisco enzyme, stomatal conductance, cell division, photosynthesis, and elongation, and it can indirectly enhance the number of leaves and flower buds [67]. Studies on lisianthus and lilium demonstrate that calcium increases the number of flowers and leaves [68,69], which agrees with the current study's results. Calcium might speed up flowering by enhancing the transport of carbohydrates from leaves [70]. Due to its connection with pectin, calcium stabilizes the cell wall, enhancing its stiffness and giving plants structural support [71]. Moreover [72], assessed the impact of foliar sprays of Ca-NPs on apple trees (*Malus domestica* Borkh 'Red Delicious'), demonstrating substantial impacts on fruit quantity and quality that were related to the Ca-NPs activity. Additionally, CaCO_3_-NPs treatments had a better impact on fruit firmness than conventional treatments (bulk), which can be attributed to the Ca-NPs size and its greater penetration, mobility, and absorption in plant tissues and cells [73].

Furthermore, compared with the control, all NPs foliar applications recorded significantly higher values at total soluble solids%, membrane stability index%, vase life, inflorescence water content, change in fresh weight, total water uptake, and water balance. In addition, all NPs foliar applications decreased the change in water loss and change in water uptake more than the control. Spraying CaCO_3_-NPs at 10 mM increased total chlorophylls and calcium contents, lignin, and cellulose% compared with the rest of the treatments. Moreover, applying SiO_2_-NPs at 3 mM increased the Si content over the other treatments. These findings could be explained by the ability of SiNPs to protect the plant against abiotic stress. Furthermore, as it could provide resistance to plant viz., SiNPs form a dual layer on the epidermal cell wall and help to reduce the negative effect of stress by modulating the antioxidant defense system [74]. In addition, the role of SiO_2_-NPs in improving the vase life of cut carnation flowers by lowering chlorophyll degradation had been recorded in another study [23]. On the contrary, the control treatment significantly increased the anthocyanin content compared with all NPs foliar treatments. These observations could be attributable to Si and CaCO_3_-NPs' beneficial effects on chlorophyll synthesis and anthocyanin content reduction via enhancing nitrogen absorption and the accumulation of aminolaevulinic acid, a precursor for chlorophyll production [75]. Likewise, due to the Si accumulation in the epidermal cells of the stem, SiO_2_-NPs have a favorable impact on the content of chlorophyll *b*. Furthermore, Si treatment increased growth and yield by enhancing plant hydration status, altering the ultrastructure of leaf organelles, triggering plant defense mechanisms, and reducing free radicals [76]. Adding Si in the apoplast of the outer walls of epidermal cells likewise induces the creation of uneven tissue on both leaf surfaces, which preempts leaf death and raises the chlorophyll content of the leaf [21]. Additionally, Si is a crucial component of the photosynthetic process, and providing plants with Si compounds raises the level of the enzyme ribulose bisphosphate carboxylase/oxygenase (rubisco) in their leaf tissue. The efficiency of carbon dioxide molecules in plant tissues is increased by this enzyme, which controls the metabolism of carbon dioxide in plants [24], and increases stomatal conductance, leaf area, and chlorophyll content [77]. Moreover, SiO_2_-NPs reduce the transpiration rate by influencing stomatal closure, increasing hydraulic conductance (water flow rate between different plant parts), reducing bacterial growth, and delaying ethylene-mediated processes like senescence [78]. Upon examining the impacts of SiO_2_-NPs on the cut branches of roses, it was found that applying silica NPs treatment increased the number of leaves and flowers, leaf chlorophyll content, flower longevity, total phenol content, and total dissolved sugars compared to the control, improving antioxidant enzyme activity and increasing flower longevity by maintaining membrane integrity, and limiting lipid peroxidation [14]. Additionally [68], stated that Si at a higher concentration (40 mg L^−1^) decreased ethylene biosynthesis, protected cells from cellular damage, decreased transpiration and evaporation rates, and increased photosynthesis, which improved solution uptake and FW of lisianthus (*Eustoma grandiflorum*) cut flowers. Moreover, silicon can speed up the production of phenol and lignin compounds as well as act as a connecting loop between lignin and carbohydrates in the cell wall which improves the cell wall strength [71,79].

Nevertheless, it has been noted in another study that gerbera in hydroponics absorbs more water at a high concentration of Ca-nano chelate at 3 g L^−1,^ and the diameter of the flower heads begins to reduce in parallel with increased water consumption because of the ligules' dehydration [80]. Researchers discovered that floral stems given CaCl_2_ and CaO-NPs at 150 mg L^−1^ maintained a weight loss of less than 6% before the 15th day of storage, demonstrating a beneficial role for CaO-NP in preserving gerbera flowers for a longer period and delaying the signs of aging or senescence [81]. CaO-NPs at 150 mgL^−1^ decreased stem bending and weight loss, improving the quality of gerbera flowers [82]. According to Ref. [80], by applying 3 g L^−1^ of Ca nano chelate, the amount of chlorophyll in gerbera plants improved. By lowering free radicals and flower senescence, Ca seems to function on the signaling pathway to stimulate the activity of the key antioxidant enzymes [83].

## 5 Conclusion

The current research aimed to study the impact of planting time and foliar application of SiO_2_-NPs and CaCO_3_-NPs at different concentrations, as well as their interaction on some pre- and post-harvest characteristics of *Dahlia pinnata* var. pinnata Cav. Vegetative and flowering characters, chemicals composition, and post-harvest parameters such as vase life and water relations, were positively affected by SiO_2_-NPs and CaCO_3_-NPs having higher values compared to the control during planting dates. Among NPs foliar applications (SiO_2_-NPs at 1.5 or 3 mM, and CaCO_3_-NPs at 5 or 10 mM), treatments of 1.5 mM SiO_2_-NPs or 10 mM CaCO_3_-NPs sprayed on dahlia plants cultured at the late planting time (20th October) had the highest values for most of the studied parameters. The findings of this investigation support the hypothesis that the suitable planting time and application of NPs as fertilizers could improve the growth and physiological status of dahlia plants.

## Author contribution statement

Mahmoud M. Kasem, Mohaned M. Abd El-Baset1, Ahmed A. Helaly, El-Sayed A. EL-Boraie,Ahmed G. Mancy, Abdulrahman Alhumaid, Mashael Daghash Alqahtani, Abdulrahman Alhashimi, Abdelghafar M. Abu- Elsaoud, Amr Elkelish and Mostafa F. El-Banna: Conceived and designed the experiments; Performed the experiments; Analyzed and interpreted the data; Contributed reagents, materials, analysis tools or data; Wrote the paper.

## Data availability statement

Data will be made available on request.

## Declaration of competing interest

The authors declare that they have no known competing financial interests or personal relationships that could have appeared to influence the work reported in this paper.
